# Supplementary material for: Knowledge, perceptions and preventive practices towards COVID-19 early in the outbreak among Jimma university medical center visitors, Southwest Ethiopia
Source: PLoS One. 2020 May 21;15(5):e0233744. doi: 10.1371/journal.pone.0233744 (PMC7241810; doi:10.1371/journal.pone.0233744)
Supplement: S1 Data — (DOCX) [file pone.0233744.s001.docx]

**Information about pre-testing of the survey questionnaire**

The following points provide the details about pre-testing of the questionnaire

1. The pretesting was conducted in same hospital few days before the survey
2. We conducted pre-testing only on 12 hospital visitors because of the urgency of the study and the World Health organization (WHO) resources were used for adapting the tool.
3. The intention of the pretesting was multiple- to ensure the questions are understandable (length, word selection, etc.)
4. To improve the understandability of the tools we accomplished the following activities
   1. Discussed with the data enumerators during the training sessions for clarity and communality across the data collectors.
   2. Role-play was demonstrated by the enumerators pair by pair, and the sub-groups have commented each other taking different scenario about the potential experiences they may face in community
   3. While on the training, we send out trainees for one day pretesting
   4. On the next day we discussed on the comments , experiences and procedures to follow while asking and responding during interview
   5. Based on pretest finding, the data collectors were received to follow similar instructions
   6. We finally reached on consensus about how to ask and help the respondents to answer to the question.
5. Based on the pretest findings we made the following improvement to the questionnaire:
   1. As much as possible the length of the questions were made shorter
   2. Instructions for how to ask and responded were added or improved to every important sections of the questionnaire
