# Supplementary material for: Knowledge, perceptions and preventive practices towards COVID-19 early in the outbreak among Jimma university medical center visitors, Southwest Ethiopia
Source: PLoS One. 2020 May 21;15(5):e0233744. doi: 10.1371/journal.pone.0233744 (PMC7241810; doi:10.1371/journal.pone.0233744)
Supplement: S2 Data — (DOCX) [file pone.0233744.s002.docx]

**Knowledge, attitudes, and practices towards COVID-19 among visitors of JUMC: Cross-sectional survey**

**Part -I Question to assess Socio-economic/back ground information**

**Instructions:** Write your response on the space provided for open ended questions and Encircle your Response for close ended questions.

| **S. No.** | Question | **Answers** |
| --- | --- | --- |
| 201 | Age | **____________** |
| 202 | Sex | A. Male B. Female |
| 203 | Religion | A. Orthodox  B. Muslim  C. Protestant  D. Catholic  E. Other____ |
| 20 | Educational status | A. unable to read and write  B. Able to read and write  C. primary /1-8th grade/  D. secondary /9-12th grade/  E. College and above |
| 206 | Marital status | A. Single  B. Married  C. Divorced  D. Widowed  E. Separated |
| 207 | Occupation | A. Merchant  B. government employer  C. Jobless  D. farmer  E. student  F. other _____ |
| 208 | monthly Income | ______________birr |

**Part II:** Questionnaire of knowledge, attitudes, and practice towards COVID-19

| **Questions** | **Options** |
| --- | --- |
| **Section I: Knowledge (correct rate, % of the total sample)** |  |
| K1. The main clinical symptoms of COVID-19 are fever, fatigue, dry cough, and myalgia. | 1. Yes 2. No |
| K2. Unlike the common cold, stuffy nose, runny nose, and sneezing are less common in persons infected with the COVID-19 virus. | 1. Yes 2. No |
| K3. There currently is no effective cure for COVID-2019, but early symptomatic and supportive treatment can help most patients recover from the infection. | 1. Yes 2. No |
| K4. Not all persons with COVID-2019 will develop to severe cases. Only those who are elderly, have chronic illnesses, and are obese are more likely to be severe cases. | 1. Yes 2. No |
| K5. Eating or contacting wild animals would result in the infection by the COVID-19 virus. | 1. Yes 2. No |
| K6. Persons with COVID-19 cannot infect the virus to others when a fever is not present. * | 1. Yes 2. No |
| K7. The COVID-19 virus spreads via respiratory droplets of infected individuals. | 1. Yes 2. No |
| K8. One way of prevention of COVID 19 is not touching the eye, nose by un washed hands. | 1. Yes 2. No |
| K9. Proper washing hand with soap and water is one method of preventing COVID-19. | 1. Yes 2. No |
| K10. Ordinary residents can wear general medical masks to prevent the infection by the COVID-19 virus. | 1. Yes 2. No |
| K11. It is not necessary for children and young adults to take measures to prevent the infection by the COVID-19 virus. * | 1. Yes 2. No |
| K12. To prevent the infection by COVID-19, individuals should avoid going to crowded places such as train stations and avoid taking public transportations. | 1. Yes 2. No |
| K13. People who have contact with someone infected with the COVID-19 virus should be immediately isolated in a proper place. | 1. Yes 2. No |
| K14. Isolation and treatment of people who are infected with the COVID-19 virus are effective ways to reduce the spread of the virus | 1. Yes 2. No |
| **Section 2: Attitudes- please respond to the following questions** |  |
| A1. Did you get health education about COVID-19? | 1. Yes 2. B. No |
| A2. Do you agree that COVID-19 will finally be successfully controlled? | 1. Yes 2. No |
| A3. Do you have confidence that Ethiopia can win the battle against the COVID-19 virus? | 1. Yes 2. No |
| A4. Do you think COVID-19 infection leads to stigma on those people who have COVID-19 infection? | 1. Yes 2. No |
| **Section 3: Practices: please tell us what you were doing to prevent COVID-19** | |
| P1. Over the last few days, have you gone to any crowded place? | 1. Yes 2. No |
| P2. Over the last few days, have you worn a mask when leaving home? | 1. Yes 2. No |
| P2. Over the last few days, do stop shaking hands of other people for greeting? | 1. Yes 2. No |
| P3. Over the last few, frequently washing hands with water and soap | 1. Yes 2. No |
| P4. Over the last few days, avoided close proximity including while greeting (within 1 meter) | 1. Yes 2. No |
| P5. Over the last few days, avoid touching eye, nose, mouth before washing hands | 1. Yes 2. No |
| P6. Over the last few days, used cover /elbow for coughing/sneezing | 1. Yes 2. No |
| P7. Over the last few days, have started to stay home | 1. Yes 2. No |
| P8. Mention and others, specify ___________________ | 1. Yes 2. No |
