# Supplementary material for: Knowledge, perceptions and preventive practices towards COVID-19 early in the outbreak among Jimma university medical center visitors, Southwest Ethiopia
Source: PLoS One. 2020 May 21;15(5):e0233744. doi: 10.1371/journal.pone.0233744 (PMC7241810; doi:10.1371/journal.pone.0233744)
Supplement: S3 Data — (DOCX) [file pone.0233744.s003.docx]

**Beekumsa, Ilaalcha, gochaa wa’ee dhibee Koronaa (COVID-19) dawwatoota hospital JUMC: Qorannoo yeroo yeroo tokkoofi geggeefamu**

**Kutaa -I Gaafii hawaasummaa fi odeefanno bu’uraa**

**Qajeelfama: Gaaffiilee armaan gadiif deebii si ilaalattu deebisi.**

| **L.G** | **Gaafii** | **Deebii** |
| --- | --- | --- |
| 201 | Umurii | **____________** |
| 202 | Saala | A. Dhiira B. Dhalaa |
| 203 | Amanata | A. Orthodoxii  B. Musliima  C. Protestantii  D. Catholicii  E. kan biraa____ |
| 20 | Barumsa | A. barreessu fi dubbisuu hin danda’u  B. bareessuu fi dubbisuu kan danda’u  C. Kutaa 1-8  D. Kutaa 9-12  E. Kolleejii fi isaa oli |
| 206 | Fuudhaa fi heeruma | A. Kan hin fuune  B. Kan fuudhe  C. Kan aadda ba’e  D. Kan irraa du’ee  E. Kan bakka biraa jiraatu |
| 207 | Hojii | A. Daldaala  B. Hojjetaa mootuma  C. Hojii-dhabaa  D. Qote bulaa  E. Barata  F. kan biraa_____ |
| 208 | Galii | ______________birrii |

**Kutaa 2 ffaa: Questionnaire of knowledge, attitudes, and practice towards COVID-19**

| **Gaafii** | **Filannoo** |
| --- | --- |
| **Garee 1: Beekumsa (deebii sirrii, % naamunaa hundaa)** |  |
| K1. Mallatolee gurguddaan dhibee COVID-19 gubaa, dadhabbii, qufaa gogaa fi maashaa caccabsuu fa’a dha. | 1. Eeyyee 2. Lakki |
| K2. Akka qufaa, dhibeen COVID-19, furri, nyaani ukkamsuu fi axxifuu baay’ee hin qabu | 1. Eeyyee 2. Lakki |
| K3. Yeroo amma dhibeen Koronaa yaalii kan hin qabnee ta’ee yeroon yoo deeggarsa argate carraan fayyuu baayee guddaa dha | 1. Eeyyee 2. Lakki |
| K4. Namni koroonaan qabame hundi mallattoo hin agarsiisu. Haa ta;u malee jaarsolii, dhibee hin daddarbine, cooma qabani irratti dhibechi ni cima. | 1. Eeyye 2. Lakki |
| K5. Bileedota alaa xuquu fi nyaachuun dhibee koronaa fi saaxiluu danda’a. | 1. Eeyyee 2. Lakki |
| K6. Namni dhibee koronaan qabame tokko yoo gubaa hin qabaanne nama biratti hin darbu* | 1. Eeyyee 2. Lakki |
| K7. Koronaan gorora xixiqa namna dhibee kanaan qabame irraan nama qaba | 1. Eeyyee 2. Lakki |
| K8.Ija, afaani fi funyaan harka osoo hin dhiqatin xuxuquu irraa of-eegguun dhibee koronaa ittisa | 1. Eeyyee 2. Lakki |
| K9. Haala gaariin saamunaa fi bishaanif dhiqachuun dhibee koronaa ittisuun ni danda’ama | 1. Eeyyee 2. Lakki |
| K10. Dhibee koronaa ittisuuf namni kamiyyuu maskii kaawwachuu ni danda’a | 1. Eeyye 2. Lakki |
| K11. Ijoollee fi dargaggoon dhibee COVID-19 ittisuu irratti hirmaachuu hin qaban * | 1. Eeyye 2. Lakki |
| K12. Dhibee koronaa ittisuuf , namootni bakka walitti qabaman fi geejjiba haawaasaa irra fagaachuun barbaachisaa dha | 1. Eeyyee 2. Lakki |
| K13. Qaamni nama dhibee koronaan qabameen waltutuqe tokko hatatamaan qofaatti addaa bafamuun ilaalamuu fi qoratamuu qaba | 1. Eeyyee 2. Lakki |
| K14. Qofaatti baasuu fi namoota dhibee COVID-19 qabamani yaaluun babalina dhibee kana ittisuuf murteessa dha | A. Eeyyee  B. Lakki |
| **Garee 2: Gaaffii Ilaalcha** | |
| A1. COVID-19 gutummaa guutuutti to’achuu dandeenyaa? | 1. Eeyyee 2. Lakki |
| A2. Barumsa waa’ee COVID-19 fudhatee jirtaa? |  |
| A3. Biyyi Itoophiyaa COVID-19 guutummatti ni balleessitee ni mo’atti? | 1. Eeyyee 2. Lakki |
| A4. Dhibeen COVID-19 loogii fi adda basuuf ni saaxila? | 1. Eeyyee 2. Lakki |
| **Garee 3: Gochaalee: mee dhibee COVID-19 hirisuu maal gochaa turtee?** | |
| P1. Guyyoota darban keessa, walga’ii deebmteetaa? | 1. Eeyyee 2. Lakki |
| P2. Guyyoota darban keessa, yeroo manaa baatu maskii kaawwatee? | 1. Eeyyee 2. Lakki |
| P2. Guyyoota darban keessa, harka qabuu dhaabdee turtee? | 1. Eeyyee 2. Lakki |
| P3. Guyyoota darban keessa, harkaa saamunaa fi bishaaniin dhiqatee? | 1. Eeyyee 2. Lakki |
| P4. Guyyoota darban keessa, walitti dhiyeeyna hiristee (1 metera) | 1. Eeyyee 2. Lakki |
| P5. Guyyoota darban keessa, ija, afan fi funyaan xuquu dhaabdee? | 1. Eeyyee 2. Lakki |
| P6. Guyyoota darban keessa, yeroo qufatuu fi axxifattu ciqileen turee? | 1. Eeyyee 2. Lakki |
| P7. Guyyoota darban keessa, mana turuu eegaletee jirata? | 1. Eeyyee 2. Lakki |
| P8. Kan biraa, adda baasi ___________________ | 1. Eeyyee 2. Lakki |
