# Supplementary material for: Knowledge, perceptions and preventive practices towards COVID-19 early in the outbreak among Jimma university medical center visitors, Southwest Ethiopia
Source: PLoS One. 2020 May 21;15(5):e0233744. doi: 10.1371/journal.pone.0233744 (PMC7241810; doi:10.1371/journal.pone.0233744)
Supplement: Attachment — Submitted filename: Response to reviewers.docx [file pone.0233744.s004.docx]

**Rebuttal letter**

**Response to reviewers**

We thank the editor and reviewers for evaluating our manuscript. We provide line by line responses to comments raised. Please, follow our responses in a yellow mark to every comment/question in green mark. We copied the review decision from submission menu of the editorial manager. We used the same letter to write our responses.

PONE-D-20-11944
Knowledge, perceptions and preventive practices towards COVID-19 among Jimma University Medical Center visitors, Southwest Ethiopia
PLOS ONE

Dear Mr. Kebede,

Thank you for submitting your manuscript to PLOS ONE. After careful consideration, we feel that it has merit but does not fully meet PLOS ONE’s publication criteria as it currently stands. Therefore, we invite you to submit a revised version of the manuscript that addresses the points raised during the review process.


We would appreciate receiving your revised manuscript by Jun 20 2020 11:59PM. When you are ready to submit your revision, log on to [https://www.editorialmanager.com/pone/](https://www.google.com/url?q=https%3A%2F%2Fwww.editorialmanager.com%2Fpone%2F&sa=D&sntz=1&usg=AFQjCNFvZ9-GTZR08pTn6lXUz6pdWQsuLA) and select the 'Submissions Needing Revision' folder to locate your manuscript file.

If you would like to make changes to your financial disclosure, please include your updated statement in your cover letter.

To enhance the reproducibility of your results, we recommend that if applicable you deposit your laboratory protocols in [protocols.io](http://www.google.com/url?q=http%3A%2F%2Fprotocols.io&sa=D&sntz=1&usg=AFQjCNFk01OGntovdK1g5gZcchbvuKd9dA), where a protocol can be assigned its own identifier (DOI) such that it can be cited independently in the future. For instructions see: [http://journals.plos.org/plosone/s/submission-guidelines#loc-laboratory-protocols](http://www.google.com/url?q=http%3A%2F%2Fjournals.plos.org%2Fplosone%2Fs%2Fsubmission-guidelines%23loc-laboratory-protocols&sa=D&sntz=1&usg=AFQjCNEu_U0-j5qqxwbP8TQOAcC7yBEtIA)

Please include the following items when submitting your revised manuscript:

- A rebuttal letter that responds to each point raised by the academic editor and reviewer(s). This letter should be uploaded as separate file and labeled 'Response to Reviewers'.
- A marked-up copy of your manuscript that highlights changes made to the original version. This file should be uploaded as separate file and labeled 'Revised Manuscript with Track Changes'.
- An unmarked version of your revised paper without tracked changes. This file should be uploaded as separate file and labeled 'Manuscript'.

Please note while forming your response, if your article is accepted, you may have the opportunity to make the peer review history publicly available. The record will include editor decision letters (with reviews) and your responses to reviewer comments. If eligible, we will contact you to opt in or out.

We look forward to receiving your revised manuscript.

Kind regards,

Wen-Jun Tu
Academic Editor
PLOS ONE

Journal Requirements:

When submitting your revision, we need you to address these additional requirements.

Please ensure that your manuscript meets PLOS ONE's style requirements, including those for file naming. The PLOS ONE style templates can be found at

[https://journals.plos.org/plosone/s/file?id=wjVg/PLOSOne_formatting_sample_main_body.pdf](https://www.google.com/url?q=https%3A%2F%2Fjournals.plos.org%2Fplosone%2Fs%2Ffile%3Fid%3DwjVg%2FPLOSOne_formatting_sample_main_body.pdf&sa=D&sntz=1&usg=AFQjCNGe6oxB056ucvyxvHe8qob9S8_XkA) and

[https://journals.plos.org/plosone/s/file?id=ba62/PLOSOne_formatting_sample_title_authors_affiliations.pdf](https://www.google.com/url?q=https%3A%2F%2Fjournals.plos.org%2Fplosone%2Fs%2Ffile%3Fid%3Dba62%2FPLOSOne_formatting_sample_title_authors_affiliations.pdf&sa=D&sntz=1&usg=AFQjCNHjeuwjv87FpHKY5ilTaf_n-Rp8PQ)

Comment:
1. Please include additional information regarding the survey or questionnaire used in the study and ensure that you have provided sufficient details that others could replicate the analyses. For instance, if you developed a questionnaire as part of this study and it is not under a copyright more restrictive than CC-BY, please include a copy, in both the original language and English, as Supporting Information. Moreover, please include more details on how the questionnaire was pre-tested, and whether it was validated.

Responses: We thank you, indeed for the advices on the revision. We have attached the questionnaires we used for the current survey. Please, check in the supplementary files. Given that this study was done urgently to inform public education and risk communication effort, we pre-tested the material on very limited people (12 hospital visitors=5% of samples). Moreover, because the questionnaire was adapted from WHO resources and other similar studies in China, we did not follow standard validation procedure than pre-testing. Please find few points in supplementary files as to how pretesting was going.

**Comment:**

2. We suggest you thoroughly copyedit your manuscript for language usage, spelling, and grammar. If you do not know anyone who can help you do this, you may wish to consider employing a professional scientific editing service.

Response: We thank you for advising us to make editions to the writing in English regarding this manuscript. We used “grammarly” online software to edit the spelling, grammar and language usage. Following that grammarly-online suggested there are word choice issues we approached PubSURE a research paper language editing service. Accordingly, there was 227 issues (with quality score of 70%=boarder line good) found on the draft manuscript. Then taking the comment reported by PubSure we cleared most of the issues, left some that were illogical. Later, in the final revised draft we got the 196/227 issues resolved. Moreover, we used a PubSURE software and we secured a certificate of quality of language that has pass score of 97%. For your information we have attached both certificates as other additional attachment with file name “Other files”. Regarding use of professional editing, we don’t afford to pay the required payment. Moreover, currently we have made changes in the manuscript in track changes and uploaded as supporting files. We uploaded a clean copy of the edited manuscript with file name *manuscript* file

Whilst you may use any professional scientific editing service of your choice, PLOS has partnered with both American Journal Experts (AJE) and Editage to provide discounted services to PLOS authors. Both organizations have experience helping authors meet PLOS guidelines and can provide language editing, translation, manuscript formatting, and figure formatting to ensure your manuscript meets our submission guidelines. To take advantage of our partnership with AJE, visit the AJE website (http://learn.aje.com/plos/) for a 15% discount off AJE services. To take advantage of our partnership with Editage, visit the Editage website ([www.editage.com](http://www.google.com/url?q=http%3A%2F%2Fwww.editage.com&sa=D&sntz=1&usg=AFQjCNHXhdBcJIwd5tNjvw3HvicptaM3-Q)) and enter referral code PLOSEDIT for a 15% discount off Editage services.  If the PLOS editorial team finds any language issues in text that either AJE or Editage has edited, the service provider will re-edit the text for free.

Upon resubmission, please provide the following:

- The name of the colleague or the details of the professional service that edited your manuscript
- A copy of your manuscript showing your changes by either highlighting them or using track changes (uploaded as a *supporting information* file)
- A clean copy of the edited manuscript (uploaded as the new *manuscript* file)

Comment:

3. Please ensure that you refer to Figure 3 in your text as, if accepted, production will need this reference to link the reader to the figure.

Response: Yes, we thank you for advising to suggest where in the manuscript figure 3 should refer to. Accordingly, you can please refer to line 184 that we now refer figure in bracket at the end of the paragraph as such, “(Figure 3)”.

[Note: HTML markup is below. Please do not edit.]

Reviewers' comments:

Reviewer's Responses to Questions

**Comments to the Author**

1. Is the manuscript technically sound, and do the data support the conclusions?

The manuscript must describe a technically sound piece of scientific research with data that supports the conclusions. Experiments must have been conducted rigorously, with appropriate controls, replication, and sample sizes. The conclusions must be drawn appropriately based on the data presented.

Reviewer #1: Yes

2. Has the statistical analysis been performed appropriately and rigorously?

Reviewer #1: Yes

3. Have the authors made all data underlying the findings in their manuscript fully available?

The [PLOS Data policy](http://www.google.com/url?q=http%3A%2F%2Fwww.plosone.org%2Fstatic%2Fpolicies.action%23sharing&sa=D&sntz=1&usg=AFQjCNEJj_X58xRcqbJNp-2AK_B_FYZKGw) requires authors to make all data underlying the findings described in their manuscript fully available without restriction, with rare exception (please refer to the Data Availability Statement in the manuscript PDF file). The data should be provided as part of the manuscript or its supporting information, or deposited to a public repository. For example, in addition to summary statistics, the data points behind means, medians and variance measures should be available. If there are restrictions on publicly sharing data—e.g. participant privacy or use of data from a third party—those must be specified.

Reviewer #1: Yes

4. Is the manuscript presented in an intelligible fashion and written in standard English?

PLOS ONE does not copyedit accepted manuscripts, so the language in submitted articles must be clear, correct, and unambiguous. Any typographical or grammatical errors should be corrected at revision, so please note any specific errors here.

Reviewer #1: Yes

5. Review Comments to the Author

Please use the space provided to explain your answers to the questions above. You may also include additional comments for the author, including concerns about dual publication, research ethics, or publication ethics. (Please upload your review as an attachment if it exceeds 20,000 characters)

Comment

Reviewer #1: The authors studied the knowledge, perceptions, and practices among Jimma University medical center (JUMC) visitors in Jimma town. They analysed 247 sampled visitors, 83% knew the main clinical symptoms of COVID-19, only 6.1% knew that children and young adults have to involve preventive measures,they concluded the status of knowledge and desirable practices were not sufficient enough to combat this rapidly spreading virus. COVID-19 risk communication and public education efforts should focus on building appropriate level of knowledge while enhancing adoption of recommended self-care practices with special emphasize on high-risk audience segments. I will give some comments as followings.
The sampled visitors was mostly young people between 20-29, and the female persons just conclude 58, this may lead to deviation, so the authors can explain the possible divation?
What precautions have been taken locally to share especially in Ethiopia?

Response: Thank you for the insight about more samples was in age groups of 20-29 years. In the first place, this is all about data. But, it is true that we noted some deviations. The following explanations can be referred to about the differences. Obviously, 40% of Ethiopian population is young people whose ages are less than 30 years old. The current study revealed 44.5% of respondents belong to this age group. Regarding, female participants the following are potential reasons for relatively low % of them sampled in this study. 1- JUMC is a specialized and referral hospital (details are mentioned about JUMC in the study setting in methods section) where many people come for services from different regions of the country, especially from Southern and Southwest region. Additionally, people come from rural settings for the services. Accordingly, it is expected that more samples can include males than females- because, in Ethiopia men are often supposed to accompany referred patients than. In Ethiopia females are supposed to stay at home in such cases because they are more important to care for children and other family members who remained at home, Moreover, obviously, sickness/injury/accidents/ are is relatively common in males and females, that means, there would be more chance for males to visit hospitals than females. In fact, the ratio of female-male is nearly 50-50 in Ethiopia. Regarding deviations, as noted in the analysis section, the effects of age and gender were already reported in ANOVA and predictors’ analysis sections. Please, refer to those sections.

Comment:
3.The style of references are no consistent, please edit it.

Response: Thank you. The reference styles are now made consistent, references # 14, 19,22, 24, and 25 (in the revised manuscript) were made consistent with others.

Comment:
4.The following references should be discussed in the revision text.
Cao JL, Hu XR, Tu WJ., & Liu Q. (2020). Clinical Features and Short-term Outcomes of 18 Patients with Corona Virus Disease 2019 in Intensive Care Unit. Intensive Care Medicine, DOI: 10.1007/s00134-020- 05987-7.
Cao JL, Tu WJ, Hu XR, & Liu Q. (2020). Clinical Features and Short-term Outcomes of 102 Patients with Corona Virus Disease 2019 in Wuhan,China. Clinical Infectious Diseases, DOI: 10.1093/cid/ciaa243/ 5814897.

Response: Thank you for providing these references we used them in discussion section, about knowledge of symptoms. and, included in the reference section. Please refer statements on 263-264 and 275-277 in discussion section of revised manuscript with track changes.

6. PLOS authors have the option to publish the peer review history of their article ([what does this mean?](https://www.google.com/url?q=https%3A%2F%2Fjournals.plos.org%2Fplosone%2Fs%2Feditorial-and-peer-review-process%23loc-peer-review-history&sa=D&sntz=1&usg=AFQjCNFLAUVWL2CBiof2AyELQ5heZ-N9BQ)). If published, this will include your full peer review and any attached files.


If you choose “no”, your identity will remain anonymous but your review may still be made public.


**Do you want your identity to be public for this peer review?** For information about this choice, including consent withdrawal, please see our [Privacy Policy](https://www.google.com/url?q=https%3A%2F%2Fwww.plos.org%2Fprivacy-policy&sa=D&sntz=1&usg=AFQjCNFbCnwIHtkhQjFNrWELvfbJHxmI2w).

Reviewer #1: No


[NOTE: If reviewer comments were submitted as an attachment file, they will be attached to this email and accessible via the submission site. Please log into your account, locate the manuscript record, and check for the action link "View Attachments". If this link does not appear, there are no attachment files to be viewed.]


While revising your submission, please upload your figure files to the Preflight Analysis and Conversion Engine (PACE) digital diagnostic tool, [https://pacev2.apexcovantage.com/](https://www.google.com/url?q=https%3A%2F%2Fpacev2.apexcovantage.com%2F&sa=D&sntz=1&usg=AFQjCNFEo2kwIg9_2n258L5WDvGur7KdjQ). PACE helps ensure that figures meet PLOS requirements. To use PACE, you must first register as a user. Registration is free. Then, login and navigate to the UPLOAD tab, where you will find detailed instructions on how to use the tool. If you encounter any issues or have any questions when using PACE, please email us at [figures@plos.org](https://mail.google.com/mail/u/0/h/1pfqtef6uts7a/?&cs=wh&v=b&to=figures@plos.org). Please note that Supporting Information files do not need this step.

Response: We have registered with PACE and all the figures do fit with PLOS. We downloaded from the PACE and uploaded as Figure 1, Figure 2 and Figures in RTF format.
